# Complex systems approaches to the adaptability of human functions and behavior in health, aging, and chronic diseases: protocol for a meta-narrative review

**DOI:** 10.1186/s13643-023-02268-4

**Published:** 2023-07-14

**Authors:** Louis Hognon, Nelly Heraud, Alain Varray, Kjerstin Torre

**Affiliations:** 1 EuroMov Digital Health in Motion, University of Montpellier, IMT Mines Ales, Montpellier, France; 2 Direction de la Recherche et de l’Innovation en Santé – Korian, GCS CIPS, Lodève, France

**Keywords:** Adaptability, Resilience, Aging, Co-morbidities, Chronic disease, Frailty

## Abstract

**Background:**

Evaluating the adaptability of human functions and behavior has become a subject of growing interest due to aging populations and the increased prevalence of chronic diseases. Various research traditions, based on complex systems theories, have addressed the adaptability of human functions and behavior. However, despite the different research traditions, no review has so far compared them to provide a reliable and useful synthetic tool. Based on an adapted methodology, study objectives are to refine common and divergent traits in the way adaptability of the human functions and behavior has been studied via complex system approaches, with a special focus on aging and chronic diseases. In order to meet this objective, we will use the methodology of the meta-narrative review, and we present in this article the protocol that we will follow.

**Methods:**

The meta-narrative review explores the contrasting and complementary ways in which researchers have studied a subject in order to synthesize information and extract theoretical and applied recommendations. In order to carry out this protocol, we detail our methodology of article extraction, coding, and synthesis. We present the six main stages of our review, from the planning stage to the recommendation stage, and the way we will implement the six principles that underpin the construction of a meta-narrative review.

**Discussion:**

The use of a meta-narrative review methodology will yield greater visibility and comprehension of the adaptability of human functions and behavior studied via complex systems-based approaches. In a broader perspective, this paper is also geared to help future researchers carry out a meta-narrative review by highlighting the main challenges encountered and anticipated as well as elements to be taken into account before starting such a project.

**Supplementary Information:**

The online version contains supplementary material available at 10.1186/s13643-023-02268-4.

## Background

Aging and related chronic conditions led to an increased percentage of the world population living with physical disabilities, multimorbidities, and an incapacity of returning to health. In this context, a growing body of research [1–6], supported by recent statements from the World Health Organization [7], has argued the need for an integrative and dynamic systems approach, to reconsider the notion of health in terms of an organism’s or individual’s capacity to adapt to disruptions. Therefore, evaluating the adaptability of human functions and behavior on various levels (neurophysiological, psychological, sensorimotor, etc.) has become a cornerstone issue in medical research and care management. The complex systems approach may provide answers since it can assess the adaptability of human functions and behavior, by capturing the interactions between the components of a system or an organism using physiological or psychological time series analyses [8–11]. However, the great heterogeneity in the way various complex systems approaches conceptualize and assess the adaptability of human functions and behavior hinders the implementation of a new vision of health based on adaptability, and its promising perspectives. Thus, one of the current challenges is to reach a common understanding of the heterogeneity of the research traditions, in order to help operationalize approaches dedicated to assessing the adaptability of human functions and behavior.

The first source of heterogeneity between the different research traditions lies in the conceptual and methodological approaches to complex systems used to evaluate the adaptability of human functions and behavior. Some authors use tools capturing fractal [12, 13] or entropic [14, 15] properties, others use physiological networks [16, 17], or the variance of these systems [18–20], with measurements either at rest or in states of disturbance of the organism. The second source of heterogeneity is inherent to the notion of adaptability itself as a research topic. Indeed, the term *adaptability* can vary (e.g., adaptability, adaptive capacity, adaptation, resilience, and flexibility), depending on several factors including disciplinary approaches (e.g., psychology, neurophysiology, and motor control), observation levels (e.g., neurological, cognitive, and sensorimotor), or research areas (e.g., fundamental or clinical research). Beyond this semantic discrepancy, the adaptability of an organism or individual is made of multiple dimensions or expression forms [21–24] (for instance, adapting positively by changing or evolving under the effect of various disruptions, or, in contrast, adapting by remaining invariant in spite of disruptions), and some research may have preferentially focused on one or another of these dimensions according to their main objective research questions. Finally, a third source of heterogeneity pertains to the specificities of the many bio-behavioral variables (e.g., variability of gait, heartbeat, and center of pressure) and target populations (e.g., elderly, healthy, with chronic diseases), which led to using the complex systems approach to assess the adaptability of human functions and behavior.

Considering this multifaceted scientific literature, our objective is to highlight commonalities and divergences in how adaptability of human functions and behavior has been studied in the past using complex systems approaches, with a special focus on aging and chronic diseases. In order to achieve this objective, it is crucial to use the type of literature review with the most appropriate data synthesis methodology. According to Pare et al. [25], one may currently differentiate nine different types of reviews, which can be grouped into four main literature review types: *data aggregation*, *critical assessment of actual literature*, *summarization of prior knowledge*, and *explanation building*. However, many of these data synthesis types are not suitable for achieving our goal. Indeed, different research traditions with different methods and concepts prevent the establishment of a standard effect, and therefore *data aggregation* is not indicated. *Critical assessment of the literature* also appears unsuitable since our present objective lies not so much in evaluating the strengths and weaknesses of different theoretical backgrounds but rather in identifying the commonalities and differences of the various scientific traditions assessing the adaptability of human functions and behavior. *Summarization of prior knowledge* comprises, on one hand, review types designed to map or describe studies rather than to compare and contrast the different research traditions, and, on the other hand, the narrative review, which is not driven by any explicit methodological requirements for the selection and analysis of the literature, thereby it could possibly induce biases and limitations in terms of transparency and reproducibility of the process.

In contrast to the previous review types, *explanation building* is concerned with finding how a set of articles supports a given hypothesis or deals with a particular topic. More specifically, the meta-narrative review sheds light on a heterogeneous field by highlighting contrasting and complementary manners in which researchers have studied the same or a similar subject [26–28], which is aligned with our objective. In addition, the meta-narrative review has been defined by a detailed, rigorous methodology promoting transparency and reproducibility, essential qualities for offering a reliable, reproducible, and useful reading of the literature. Consequently, the methodology of the meta-narrative review seems the most appropriate for highlighting how different traditions of research have studied the adaptability of human functions and behavior via complex systems approaches, with a special focus on aging and chronic diseases. Hereafter, we present the specific way to implement this protocol in light of this topic.

## Methods/design

The meta-narrative review method has recently been developed for synthesizing information. It is designed to analyze the way a topic has been addressed by different research traditions [26]. This methodology is underpinned by six principles [27], namely *pragmatism*,* pluralism*,* historicity*,* contestation*,* reflexivity*, and *peer review*, constituting the essence of meta-narrative reviews regardless of their topic, promoting fair and weighty narrative accounts of multifarious research traditions on a subject. These principles are individually defined in Table 1 and contextualized for the framework of our project.

**Table 1:** Principles of the meta-narrative review

Principles	Definitions	Application
Pragmatism	The researcher is guided by the needs of the intended audience and what is most likely to raise awareness [29].	Part of the literature currently promotes a paradigm shift to re-consider the notion of health in terms of an individual or organism’s adaptive capacities, notably using concepts and tools of complex systems approaches. However, this paradigm shift is associated with heterogeneity in complex systems approaches, likely to limit the impact in research communities not used to such approaches. A review highlighting the commonalities and divergences in the way the adaptability of human functions and behavior has been studied by complex system analyses should help promote the operationalization of adaptability as a new vision of health
Pluralism	The subject is explored from multiple angles and the quality of the research or evidence is judged by criteria intrinsic to the research tradition from which it has emerged. The aim is “to expose tensions, map diversity and communicate the complexity of how different traditions contribute to understanding the problem” [29].	Preliminary search and mapping have revealed that several research traditions based on complex system approaches coexist in the literature. This great plurality of approaches already led us to a pre-screening to establish a research strategy to only include those articles that deal with the evaluation of the adaptability of human functions and behavior. In parallel, co-authors have different scientific backgrounds and pragmatic approaches favoring a more holistic view of the subject and it would therefore prevent from focusing on a single research tradition
Historicity	The subject is explored by different research traditions as they develop over time, including major events, key scientists, and the findings that shaped the tradition [29].	Research traditions using a complex systems approach have emerged at different times and in different disciplines, building over time on different methods and concepts. However, some of these approaches recently converged towards the objective of assessing the adaptability of human functions and behavior in relation to health conditions. This is where the historical aspect will be of interest to us, and we will assess when and how these approaches focused on this objective, highlighting temporal disparities and landmarks in their respective evolutions
Contestation	Conflicting data’ from different research traditions should be examined to generate higher-order insights (for example, about how different research teams framed the issue differently or made different assumptions about the nature of reality) [29].	Based on the information collected, we will place each research tradition in its conceptual and methodological framework, in order to refine where divergences between traditions can emerge (e.g., the fractal approach is associated with the concept of self-similarity and long-term correlation and is evaluated through tools quantifying the self-similarity of a physiologic time series). Then we will relate the different traditions to the notion of adaptability of human functions and behavior. Subsequently, we will identify, if any, the conflicting data between these traditions (i.e., what definitions of adaptability or experimental approach are used ….), but also complementary data in the event that certain data do not appear contradictory but rather provide similar information, but are dependent on the conceptual and methodological framework, for example. This will enable to reason about simple differences or convergences, exposing them as they are, but also to understand why these differences exist in regards to the conceptual and theoretical frameworks of each research tradition. These analyses will be brought to light in the synthesis phase in order to understand how several research traditions fit into the objective of evaluating the adaptability of human functions and behavior
Reflexivity	Researchers should continually reflect, individually and as a team, on the conclusions that emerge throughout the review [29].	We have and will continue to reflect by constantly interacting throughout the preparation, planning, construction and writing of the meta narrative review. During the first period of preparation and planning, the team was able to learn the methodology of meta-narrative review and its principles, as well as assimilating the problem being addressed. This allowed us to develop a reflective and critical mindset in which it was possible to question our approach and later our selection, coding and synthesis of information. Moreover, as the procedure of the meta narrative review is essentially non-linear, we went back and forth between certain stages, questioning each time the relevance of our decisions in the light of certain repercussions becoming visible at the subsequent stage. Finally, the multidisciplinary nature of our team is an asset for relevance, capacity for criticism and reflection as well as ensuring sound scientific judgments, which will be at the core of this review
Peer review	New results are tested by presenting them to external reviewer in a formative way, and this feedback feeds into further reflection and analysis [29].	We will ask several scientists, specialized in complex systems, chronic diseases and health, outside of our research team, to analyze our synthesis phase in order to check if we yielded the differences and complementarities between the various research traditions while respecting the principles of historicity, pluralism and contestation. Finally, we will present our recommendation phase to an external evaluator to ensure that it is consistent with the data extracted from the articles and the synthesis phase. The objective will be to nourish our reflection in the light of the evaluators’ feedback to ensure a coherent and structured narrative account of the collected information

While these principles are intended to guide authors through the whole journey of writing the meta-narrative review, the process itself is made up of six building phases: the *planning phase,* the *searching phase,* the *mapping phase,* the *appraisal phase,* the *synthesis phase,* and the *recommendation phase* [26]. These phases organize the work logistics for the project team, i.e., selecting, extracting, evaluating, and synthesizing the collected data, as well as establishing recommendations based on the entire work. Aims and case-specific contents of each phase are detailed below, but already it is worth noticing that the methodology of the meta-narrative review is not linear, indeed going back and forth between the different phases is necessary for its construction.

In order to develop and facilitate the development and communication of this protocol we followed the guidelines of The Preferred Reporting Items for Systematic Review and Meta-Analysis Protocols (PRISMA-P) (see Additional file 1). The protocol described in this article is registered with PROSPERO [CRD42021236736], the International Prospective Register of Systematic Reviews.

### Planning phase

The first phase of a meta-narrative review is to establish a team and define the role of each team member with regard to the project direction. In our case the team is multidisciplinary, with a specialist in complex systems (KT), a specialist in health and physiology (AV), and a specialist in clinical research (NH), working together for the purpose of LH’s thesis. In parallel, during the planning phase, we exchanged with different scientists who either worked on the creation of the meta-narrative review methodology or already carried one out, in order to refine our understanding of this new type of systematic review and avoid certain pitfalls. This phase, which is now completed, lasted about 3 months and required several meetings to acquire the basis of the meta-narrative review methodology, and jointly establish the direction of the project. At the beginning, our team held several meetings to discuss the relevance of the subject and the precise scope of our review, the timely opportunity in light of the literature. Observing at the same time the need expressed in the literature on aging and chronic diseases to consider the notion of health as the ability to adapt, and the resurgence or emergence of research traditions that proposed to assess adaptability via complex systems approaches without systematic relationships between them, gave us reasonable confidence in the need to establish a common understanding of this topic. Afterwards, LH led the work to determine the questions of the meta-narrative review and wrote the protocol. LH also familiarized the other team members with the meta-narrative review approach and methodology. At the end of this first phase, the respective roles and responsibilities of each team member were determined for the subsequent phases.

### Searching phase

The second phase of the meta-narrative review, the searching phase, consists in developing an article search methodology to extract the maximum number of articles related to the review topic This phase encloses two essential steps: the methodology for researching articles and their selections based on defined criteria. Before starting this phase, a non-exhaustive compilation of the literature on the adaptability of human functions and behavior was carried out by LH, to facilitate a preliminary identification of both the different research traditions via several key articles, and the variety of terminologies used to refer to the review topic according to research fields.

The first step of the searching phase was to create different groupings of terms in order to be able to combine them for the most efficient search. Through reading the articles compiled by LH, different terms and keywords were picked up during meetings and then grouped into three categories: the first corresponds to the terminologies used to refer to the adaptability of human functions and behavior, such as *resilience*, *physical resilience*, *adaptability*, and *adaptive capacities*. The second corresponds to the populations targeted for our research, typically including individuals over 18 years of age, elderly populations, and people suffering from chronic diseases. The third category grouped the different concepts and methodologies specific to complex systems approaches, such as nonlinear dynamics, fractals, entropy, and resilience. In order to reduce the number of terms within each of these categories, we retained MeSH Terms where relevant.

Before carrying out the search on all the databases, a first search was performed on PubMed to ensure that the combination of the three search categories yielded relevant results, but also to identify different off-topic terms. Indeed, due to the fact that terms related to the adaptability of human functions and behavior are used in a lot of different research areas (e.g., economy, climate, urbanization, and management), a list of terms to be excluded was constituted and grouped with MeSH Terms if possible. Once we refined the three search families and list of terms to exclude, we proceeded to search for articles in the following databases: PubMed, Web of Science, and Science Direct. The electronic search strategy included a combination of Boolean operators and MeSH terms formulated according to the characteristics of each database. Qualitative, quantitative, and mixed methods studies up to January 2021 were eligible, articles such as personal narratives, conferences, editorials, or books (see Additional file 2) were excluded. A complementary search phase will consist in making a reference follow-up within the selected articles, in order to yield unidentified complementary articles. Finally, the articles that team members were aware of but that were not captured by the search processes were added later, and also submitted to the screening process detailed below. All selected articles were imported and managed using the Zotero citation manager.

The second step of the searching phase is screening the articles collected during the first phase based on exclusion criteria, to select only the most relevant to our analysis. Before starting the screening process, all duplicates were removed. In order to determine the exclusion criteria, a sample of articles was selected from those retrieved from the databases, and each team member had to determine why certain articles should be excluded. After several meetings, we were able to determine the main exclusion criteria that allowed us to easily exclude off-topic articles (e.g., climate, war, robotics, finance, forest), articles not addressing human functions and behavior, humankind or involving subjects under 18 years of age, articles addressing adaptability but not from a complex systems approach. Nevertheless, some articles for which we had doubts were provisionally kept to determine their eligibility during a meeting specifically dedicated to that task. After that, the screening process will take place in two phases, first on title and abstract reading, and second on full-text reading. At each phase, all abstracts/articles will be read and selected by LH. In each phase, at 25, 50, 75, and 100% of the total number of abstracts/articles treated by LH, the other team members will carry out a monitoring based on a sample of articles, equivalent to 10% of the total number of abstracts/articles, to ensure screening consistency. We set the screening consistency threshold at 80%; below that level, a new monitoring will be required on another sample. In the event of a conflict or doubt about the inclusion or exclusion of an article, LH will ask a team member for advice. If the problem persists, a meeting will be scheduled with all team members. Reasons for the exclusion of certain articles will be specified at each phase and collected in a spreadsheet that will be provided during the final publication of the meta-narrative review.

### Mapping phase

The third step of the meta-narrative review consists in extracting the most relevant information from the selected articles, in order to reply to the questions of the review and identify the research traditions and their characteristics. According to Wong et al. in 2013 [26], a meta-narrative analysis should answer the following questions: What research traditions have addressed this broad subject? How has each tradition conceptualized the subject? What theoretical and experimental approaches have been used? What are the main empirical results? What lessons can be learned by combining and comparing the results of different traditions? We considered these general recommendations in conjunction with the state of our a priori research knowledge on our topic, to define a matrix with 6 dimensions of information to encode the selected articles. This encoding should provide us with the necessary information to determine which different research traditions have conceptualized and studied the adaptability of human functions and behavior through complex systems approaches and how they did it. The first dimension relates to the global *identification of the research traditions*: (i.e., research traditions based on complex systems approaches like the loss of complexity, network physiology, resilience, physical resilience, or others). The second dimension refines whether the notion of adaptability constituted the *primary focus of the article*, (i.e., adaptability is the main variable of interest or the central theme of the article), or whether it appears as a *secondary consideration* (i.e., adaptability is merely mentioned as a subsidiary notion, for example as a perspective in the discussion. The third dimension pertains to the *definition of the adaptability of human functions and behavior*, when provided*.* The fourth dimension is to specify the *approach used*, whether theoretical or experimental. In the case of an experimental study we will further collect the main signal analyzed (e.g., heart and respiratory rates, stride times, tapping, and center of pressure), methods of analysis, the population studied, and main results. The fifth dimension explores whether the study had *an applied or a theoretical objective*, or both. Finally, the sixth dimension determines the *kind of interpretative approach* taken in the article. More precisely, we will distinguish between three kinds of interpretative approaches: articles with a predominant mechanistic scope, meaning with a vocation to find causal chains of explanatory mechanisms to understand the results (e.g., an article analyses markers of sympathetic and parasympathetic activity in the variability of the cardiac system, in order to understand the link between the activity of the autonomic system and the adaptability of human functions and behavior); articles with a predominant systemic scope, meaning results are dealt within a theoretical framework assuming that given properties are the universal product of complex adaptive systems regardless of their precise nature (e.g., the loss of adaptive capacity in individuals with chronic conditions compared to healthy subjects would tend to be explained by previous results). Finally, articles with a predominant idiographic scope, meaning the results are merely considered within the specific case of the study, without attempting to propose generalizable explanations, or remaining descriptive (e.g., a lower level of complexity of heart rate variability, in individuals with pathologies compared to healthy subjects, suggests a loss of adaptive capacity. This 6-dimensional matrix was tested on a sample of articles to check for relevance and utility. Several corrections were necessary, further underlining the non-linear aspect of the meta-narrative review methodology. LH will perform the encoding of articles, and monitoring phases at 25, 50, 75, and 100% of the total number of encoded articles will be carried out by the other members of the team, AV, KT, and NH, to ensure encoding consistency. Nevertheless, monitoring in this mapping phase will not be accompanied by a consistency threshold as in the screening phase. Upon the final release of the meta-narrative review, the data extraction spreadsheet will be provided.

### Appraisal phase

The appraisal phase must make it possible to ensure that identified and selected articles are both relevant to the focus of the study and bear a rigorous scientific method. Due to the heterogeneity of articles on the adaptability of human functions and behavior assessed via complex systems approaches, it was considered counterproductive to use an evaluation tool for the quality of articles included and encoded. Therefore, the quality of articles will be discussed during several steps: first throughout the selection and encoding phases, second when reading the full text, third in several meetings, and finally during the ongoing collegial dialog between team members. This work will allow for continuous methodological adaptation and is consistent with the iterative process of the meta-narrative review [26, 27]. Articles excluded based on a poor quality score will be recorded in an annex, and the reason for exclusion will be mentioned.

### Synthesis phase

The synthesis phase corresponds to the phase of aggregation and analysis of the information extracted from the articles, in order to answer the questions of the meta-narrative review and to develop a coherent and representative narrative of the adaptability of human functions and behavior assessed through the complex systems approaches. It will be based on several analysis techniques in accordance with the meta-narrative review methodology: paradigm bridging (identifying commonalities), paradigm bracketing (exploring differences), interplay (examining and explaining tensions or contradictions in the data), and meta-theorizing (exploring patterns that cut across different understandings), and takes place in 3 steps. First, data collected during the mapping phase are aggregated during an analysis phase, to help provide a representation of different research traditions. Second, according to Greenhalg et al. [27], the synthesis phase is interpretative in the sense that compares and contrasts the research traditions and methodological approaches used. Therefore, differences and conflicts within and between research traditions have to be interpreted in order to elicit an explanation of the findings. For example, we will examine how and why the notion of adaptability of human functions and behavior is defined and explained differently in different research traditions by taking into account their respective scientific background. Third, we will add an interpretation of the data collected in order to establish how we can jointly understand and use these different research traditions from a theoretical and an applied perspective, thereby also identifying the main gaps in the research on the adaptability of human functions and behavior to indicate where research could be promisingly directed. These three steps will emerge from several team meetings on the results and information collected. Once this synthesis phase is completed, team members will meet to discuss the relevance and robustness of the narrative employed. If the team has doubts about its relevance and robustness, other experts on the topic will be invited to read and review the summary part.

### Recommendation phase

The final step of the meta-narrative review is to establish policy, practice, and research recommendations. The objective of considering the notion of health as the adaptability of the human functions and behavior is an important health issue, but does not yet have a political aim as such. Therefore, the recommendations of this systematic review will focus on research practices and scientific directions. More precisely, they will attempt to provide guidance on how these different research traditions can be used to understand the adaptability of human behavior and whether it is possible to combine them according to their theoretical and methodological conceptions. In addition, theoretical and methodological concerns will be raised about how to conceive an association between these research traditions and the adaptability of human functions and behavior. Finally, medical orientations on the use of these research traditions for aging and chronic disease populations, such as health status prediction, monitoring, and management, will be provided.

## Discussion

Apprehending the adaptability of human functions and behavior with complex systems approaches has become a subject of expanding researches with a common objective to address challenges inherent to aging and chronic diseases. However, the plurality of approaches, definitions employed, observation levels, contexts, and methodologies (and especially variables used) leave a blurry picture of the state-of-the-art, thereby hampering the implementation of promising perspectives for assessing the adaptability of human functions and behavior. Thereby, our objective is to highlight the commonalities and divergences in the way the global notion of adaptability of human functions and behavior has been studied by complex system analyses, with a special focus on aging and chronic diseases. In order to achieve this, we determined that the methodology of the meta-narrative review is the most suitable, and are presenting the protocol that we are going to follow. This procedure ensures the most transparent approach and constitutes an opportunity for data sharing to promote future meta-narrative reviews. Thus, within this discussion, we address the main challenges already encountered and those that we anticipate during the course of the review.

The main challenge encountered so far concerns the search and selection of articles. Indeed, within the framework of a meta-narrative review, the objective is to explore different research traditions and therefore yield from the database search all main relevant articles. For our review, the terms *adaptability* or *resilience*, for example, are widely cited in numerous scientific specialties, increasing the occurrence of off-topic articles in the early selection stages. As a result, we proceeded to multiply the analyses of sample articles retrieved from the databases, to determine the main relevant terms to be excluded, while retaining relevant articles. Nevertheless, off-topic articles still remained after the searching step, and it was therefore crucial to determine the most adequate exclusion criteria for the selection step, using the pragmatism principle. Indeed, as pointed out by Newman et al. in 2018 [30], pragmatism, in the meta-narrative review indicates that it is not easy to select articles when the literature on a subject is not contained and delimited [30]. To overcome this challenge, based on the article title and abstract reading, we opted for a benefit-of-the-doubt approach: we created exclusion criteria allowing a certain permeability for studies containing terms associated with the notions of complex systems or adaptability, without necessarily being directly relevant for our work. These articles were then grouped together and examined again, one by one, during a meeting with all team members to decide whether or not to include them.

Other challenges identified are related to two of the meta-narrative review principles (Table 1). The first relates to the principle of pluralism through the identification and exploration of the different research traditions. The aim is to determine which research traditions have studied the adaptability of human functions and behavior with a particular focus on aging and chronic diseases, without highlighting those that are familiar to us. To achieve this, we constituted a multidisciplinary team, with researchers specializing in complex systems approaches, health and physiology, and clinical research, allowing for complementary and new visions with moderate a priori considerations regarding each other’s field of interest. In addition, we will use a peer review process to submit our results to researchers outside the team, who are specialized in complex systems, chronic diseases, and health, in order to obtain the maximum number of feedbacks. Finally, in light of the multiple research traditions based on complex systems approaches covering various research topics, we carried out a pre-screening phase and created a specific search methodology on the different databases (see Additional file 2) in order to include only those articles related to the adaptability of human functions and behavior.

The third challenge relates to the principle of contestation, with the exposure and understanding of the contradictory data within and between research traditions. However, this principle can be difficult to respect if research traditions do not contradict one another. For example, even when using different models and methods, studies may present their results or theoretical concepts regarding the adaptability of human functions and behavior on a common — notably systemic — interpretative level. Conversely, studies using the same analysis method in the same topic may take different and not always compatible, nor even comparable, interpretative approaches. Therefore, it is not only contestation that will matter, but also a common understanding of these traditions with their different kinds of interpretative approaches, and their respective validity perimeters. The meta-theorizing method can link studies to their background and theoretical models, to better understand divergences and similarities in the conception of the adaptability notion.

Finally, it seems worth highlighting the time-consuming nature of a meta-narrative review as a factor to be taken into consideration. The time required for appropriation and application of the methodology may exceed several months, with phases of search and selection of articles being extremely time-consuming. Therefore, it is necessary to ensure the availability of team members, over several months, and to distribute tasks accordingly. Finally, taking into account the subjectivity inherent in systematic reviews [31], we have published the protocol in PROSPERO, and we will make sure, for the sake of transparency, that it is updated should there be changes or amendments.

To our knowledge, this review constitutes the first systematic review to synthesize the different research traditions that have used complex systems approaches to study the adaptability of human functions and behavior. The resulting synthesis will be the completion of a rigorous methodology guided by the principles of meta-narrative review in order to provide a common understanding of the complex systems approaches and to promote the investigation of the adaptability of human functions and behavior as a valuable conception of health.

## Supplementary Information

**Additional file 1.** Prisma-P checklist.

**Additional file 2.** Search strategy.

## Data Availability

The data supporting the conclusions of this article will be made available by the authors, without undue reservation.
