# Supplementary material for: Complex systems approaches to the adaptability of human functions and behavior in health, aging, and chronic diseases: protocol for a meta-narrative review
Source: Syst Rev. 2023 Jul 14;12:122. doi: 10.1186/s13643-023-02268-4 (PMC10347848; doi:10.1186/s13643-023-02268-4)
Supplement: Supplementary file 1 — Additional file 1. Prisma-P checklist. [file 13643_2023_2268_MOESM1_ESM.pdf]

Additional File 1

PRISMA-P 2015 checklist

ADMINISTRATIVE INFORMATION

Title

**Identification**		Complex systems approaches to the adaptability of human functions and behaviour in healthy, aging, and chronic diseases: a meta-narrative review
**Update**	Item 1b	This is an original review, not an update of a prior review.
**Registration**	Item 2	PROSPERO [CRD42021236736]

Authors item 3a

Louis Hognon, (corresponding author)
Phd student
University of Montpellier
EuroMov Digital Health in Motion, University of Montpellier, IMT Mines Ales, Montpellier, Montpellier, France
louis.hognon@umontpellier.fr

Professor Alain Varray
University of Montpellier
EuroMov Digital Health in Motion, University of Montpellier, IMT Mines Ales, Montpellier, Montpellier, France
alain.varray@umontpellier.fr

Dr Kjerstin Torre
University of Montpellier
EuroMov Digital Health in Motion, University of Montpellier, IMT Mines Ales, Montpellier, Montpellier, France
kjerstin.torre@umontpellier.fr

Ms Nelly Heraud,
Director of research team, Korian
Lodeve, France
nelly.heraud@korian.fr

Contributions item 3b

LH determined the focus and questions of the meta-narrative review and wrote the protocol. LH also familiarized the rest of the team with the meta-narrative review approach and its methodology. The rest of the team, AV, KJ and NH helped redefine the questions and finalize the protocol.

**Amendments**	Item 4	The Research Coordinator (LH) will document and report any amendments to the protocol.
**Support**		
**Sources**	Item 5a	No one
**Sponsor**	Item 5b	No one

INTRODUCTION

Rationale Item 6

The evaluation of the adaptability of human functions and behaviour has become a growing subject of interest, due to the increase in aging and chronic diseases populations. Several distinct research traditions, based on the theory of complex systems, study the adaptability of human functions and behaviour. However, there is no review to synthesize and compare these different research traditions. Therefore, our objective is to highlight the commonalities and divergences in the way the notion of the adaptability of human functions and behaviour has been studied by complex system approaches, with a special focus on aging and chronic diseases.

Objectives Item 7

Our research questions are:

1. What are the research traditions that have studied the subject of adaptability of human functions and behaviour in chronic conditions from a complex systems approach?

(human functions and behaviour defined as anything that a human organism does involving action and response to stimulation)

2. How have research traditions studied this subject?
3. What are the commonalities and differences between these traditions?
4. How can these traditions be commonly understood on the levels of theoretical conception and applied perspectives?

METHODS

Eligibility criteria Item 8

Eligibility: all published academic articles on the adaptability of human functions and behaviour studied with complex system approach. Thesis, letters, editorials, conference abstracts, poster presentations and dissertations are excluded.

Exclusion criteria

- Abstract unavaible
- Type of articles excluded (eg : conference, editorial, book)
- Off-topic articles (eg: climate, war, robot, finance, forest)
- Articles no related to human kind or human under 18 years' old
- Articles not using time series analysis or which talking about adaptability without using a complex systems approach

Information sources Item 9

The search period is 1900 – January 2021.

The search includes 3 academic databases:

- PubMed
- ScienceDirect (Elsevier)
- Web of Science (Science Citation Index, the Social Sciences Citation Index) (ISI)

Search strategy Item 10

The article search is carried out by two processes. The first is a database searching on Pubmed, Web of Science and Science Direct, and the second is tracking references from key articles selected. The articles that the team members are aware of but that will not be captured by the search processes will be added later, and also submitted to the screening process. The database searching strategy includes a combination of Boolean operators and MeSH terms that are formulated according to the characteristics of each database, defining three distinct groups of requested terms and one group of terms to be excluded. The group of terms to be excluded has been established through preliminary searches, and describes the different research topics that we wish to exclude in order to focus on the subject of the adaptability of human functions and behaviour in chronic conditions from a complex systems approach. Below, is an example of the database search strategy on Pubmed.

#1: Adaptation, psychological [MeSH Terms] OR Adaptation, physiological [MeSH Terms] OR Adaptation, biological [MeSH Terms] OR General Adaptation Syndrome [MeSH Terms] OR Emotional Adjustment [MeSH Terms] OR Homeostasis OR Physical resilience* OR Stability* OR Plasticity* OR Robustness* OR Biological evolution [MeSH Terms]

#2: (Health [MeSH Terms] OR Aging OR Elderly OR Aged OR geriatrics OR Disease OR Chronic Health Evaluation* OR Physiopathological* OR psychomotor performance [MeSH Terms] OR Mortality [MeSH Terms] OR Frail Elderly [MeSH Terms]) AND (Humans [MeSH Terms])

#3: Systems Analysis [MeSH Terms] OR Nonlinear Dynamics [MeSH Terms] OR Fractals [MeSH Terms] OR Stochastic Processes [MeSH Terms] OR Biological Variation, Population [MeSH Terms] OR Time series analysis* OR Resilience* OR Entropy*

#4: Economic* [MeSH Terms] OR Natural Disasters [MeSH Terms] OR Geological Phenomena [MeSH Terms] OR Climatic Processes [MeSH Terms] OR Environment [MeSH Terms] OR Environmental Pollution [MeSH Terms] OR Amino Acids, Peptides, and Proteins [MeSH Terms] OR Humanities [MeSH Terms] OR Dental Health Services [MeSH Terms] OR Epidemics [MeSH Terms] OR Pandemics [MeSH Terms] OR COVID-19 [MeSH Terms] OR MicroRNAs / genetics* OR Nurses [MeSH Terms] OR Caregivers [MeSH Terms] OR Self Care[MeSH Terms] OR Stress Disorders, Traumatic [MeSH Terms] OR Population [MeSH Terms] OR Leadership [MeSH Terms] OR Chemistry [MeSH Terms] OR Homosexuality* OR Bisexuality* OR lesbian* OR Transgender Persons* OR gay* OR Urban Health Services [MeSH Terms] OR Socioeconomic Factors OR Sociological Factors [MeSH Terms] OR Social Behavior* OR Social Work* OR Social Problems [MeSH Terms] OR Social Environment [MeSH Terms] OR Refugees [MeSH Terms] OR Technology and Food and Beverages Category [MeSH Terms] OR Image Processing, Computer-Assisted [MeSH Terms] OR Culture [MeSH Terms] OR Radiographic Image Interpretation, Computer-Assisted [MeSH Terms] OR Image Interpretation, Computer-Assisted [MeSH Terms] OR Organization and Administration [MeSH Terms] OR Plants [MeSH Terms] OR Fungi [MeSH Terms] OR Interviews as Topic [MeSH Terms] OR Interview* OR Animal* OR Surveys Questionnaires* OR School Health Services [MeSH Terms] OR Education [MeSH Terms] OR Ergonomics [MeSH Terms] OR Osteopathic Physicians [MeSH Terms] OR Art Therapy [MeSH Terms] OR Nose / surgery* OR Weightlessness [MeSH Terms] OR Delphi Technique [MeSH Terms] OR Learning [MeSH Terms] OR anatomy histology* OR Veterinarians [MeSH Terms] OR Students, Health Occupations [MeSH Terms] OR Infant [MeSH Terms] OR Child [MeSH Terms] OR Child Development* OR Adolescent [MeSH Terms] OR Neoplastic Processes [MeSH Terms] OR Military Personnel [MeSH Terms] OR Bone and Bones [MeSH Terms] OR Internal-External Control [MeSH Terms] OR Rotation [MeSH Terms]

((#1) AND (#2) AND (#3)) NOT (#4)

Filters: All years; language: English; Species: Human; Article Type included: Case Reports, Clinical Study, Clinical Trial, Comparative Study, Controlled Clinical Trial, Corrected and Republished Article, Dataset, Evaluation Study, Guideline, Historical Article, Journal Article,

Meta-Analysis, Multicenter Study, Practice Guideline, Pragmatic Clinical Trial, Randomized Controlled Trial, Review, Systematic Review, Twin Study, Validation Study

Study records

Data management Item 11a

Reference management with Zotero.

Selection process Item 11b

Studies will be selected if they do not validate one of the established exclusion criteria. Two phases of selection will be carried out, first on the title and abstract and second on the full text. At each phase, all articles will be read and selected by LH. The other members of the team will carry out monitoring phases at 25, 50, 75 and 100% of the total of articles at each phase on a sample, to ensure screening consistency which must be at least equal to 80%. If this threshold is not obtaining, a new monitoring will be done on an another sample. In the event that there is a conflict or doubt about the inclusion or exclusion of an article, LH will seek a member of the team. If the group member is unable to resolve the problem, then a meeting will be scheduled with all team members to resolve it.

Data collection process Item 11c

A spreadsheet containing 6 categories to extract the information was created in order to know which and how different research traditions have conceptualized and studied through complex systems approaches, the adaptability of human behaviour.

1. The identification of the research tradition (fractal, entropy, network physiology, DIOR's, others).
2. The definition used to talk about the adaptability of human behaviour.
3. The focus of the article on the adaptability of behaviour. Either, is it primary, adaptability is a variable or the central theme of the article or secondary, like by saying that this method or this result reflects adaptability.

4. The approach used, was it just theoretical or experimental. If the study was experimental then we collect the method and signal used, the population studied, and the main result.
5. The objective: applied or theoretical
6. The interpretative level of the article, either by declaring that it was mechanistic with a vocation to find one or more explanatory mechanisms, or systemic by suggesting hypotheses, previous results in the literature to illuminate, describe and explain results, or idiographic if the article focuses solely on its subject of study and does not attempt to find universal laws.

Data items Item12

Data categories are anticipated to include main research traditions, their concepts, theories, methods, research questions, and evidence.

Outcomes and prioritization Item 13

Articles addressing any of the research questions will be mapped by research tradition, with particular attention to contestations, conflicting findings, and discordant conclusions.

Risk of bias in individual studies Item 14

Due to the heterogeneity of articles on the subject of adaptability of human behaviour apprehended with complex systems approaches, it was considered counterproductive to use an assessment tool on the quality of the articles included and encoded. The quality of the articles will be discussed throughout the selection and encoding phase upon reading the full text, over several meetings and a continuous and collegial dialogue between team members. This work will allow for continuous adaptation, and is consistent with the iterative process of the meta-narrative review.

Data synthesis

Items 15 a, b, c No quantitative synthesis is planned.

Item 15 d

The data synthesis will be based in accordance to the meta-narrative review methodology: paradigm bridging (identifying commonalities), paradigm bracketing (exploring differences), interplay (examining and explaining tensions or contradictions in the data), and meta-theorizing (exploring patterns that cut across different understandings) and will take place in 3 steps :

- First, the data is aggregated during an analysis phase and helps providing a representation of different research traditions.
- Second, according to Greenhalg et al. 2005 (26), the synthesis phase is interpretative in the sense that one must compare and contrast the research traditions and the methodological approaches used. Therefore, the differences and conflicts within and between research traditions must be interpreted in order to elicit an explanation of the findings made.
- Third, we will add an interpretation of the data collected in order to establish how we can jointly understand and use these different research traditions from a theoretical and an applied perspective. These three steps will be the culmination of several meetings among team members about the results and information collected. When this synthesis phase is completed, team members will meet to discuss the relevance of the narrative discourse employed and the robustness of explanations of understanding between research traditions

Meta-bias(es) Item 16

No one reported

Confidence in cumulative evidence Item 17

The synthesis will be evaluated with the training materials created by Greenhalgh and Wong.

http://www.ramesesproject.org/media/Meta_narrative_reviews_training_materials.pdf
