# Supplementary material for: Complex systems approaches to the adaptability of human functions and behavior in health, aging, and chronic diseases: protocol for a meta-narrative review
Source: Syst Rev. 2023 Jul 14;12:122. doi: 10.1186/s13643-023-02268-4 (PMC10347848; doi:10.1186/s13643-023-02268-4)
Supplement: Supplementary file 2 — Additional file 2. Search strategy. [file 13643_2023_2268_MOESM2_ESM.pdf]

Additional File 2

Search Strategy

Title : Complex systems approaches to apprehend the adaptability of human functions and behaviour with a special focus on healthy aging and chronic conditions: a meta narrative review

Authors: Louis Hognon [1] *, Nelly Heraud [2], Alain Varray [1], Kjerstin Torre [1]

Affiliation : [1] EuroMov Digital Health in Motion, University of Montpellier, IMT Mines Ales, Montpellier, France ; [2] Direction de la Recherche et de l'Innovation en Santé – Korian

*** Correspondence:**

Louis Hognon
EuroMov Digital Health in Motion, University of Montpellier, IMT Mines Ales
700 Avenue du Pic Saint-Loup 34090 Montpellier

Email: louishognon@outlook.fr

Phone number: +33 (0)6.50.33.85.49

- **Procedures**

The electronic research of articles was carried out on the following databases: Pub Med, Science Direct and Web of Science, with combinations of keywords, MeSH terms and Boolean operators as well as the application of various selection filters according to the possibilities and the characteristics of databases. The searches for each database are specified below and have been made with # :

#1 = terms related to the adaptability of the human behaviour

#2 = populations studied (old, chronic disease)

#3 = complex system approaches used

Pub Med

#1: Adaptation, psychological [MeSH Terms] OR Adaptation, physiological [MeSH Terms] OR Adaptation, biological [MeSH Terms] OR General Adaptation Syndrome [MeSH Terms] OR Emotional Adjustment [MeSH Terms] OR Homeostasis OR Physical resilience* OR Stability* OR Plasticity* OR Robustness* OR Biological evolution [MeSH Terms]

#2: (Health [MeSH Terms] OR Aging OR Elderly OR Aged OR geriatrics OR Disease OR Chronic Health Evaluation* OR Physiopathological* OR psychomotor performance [MeSH Terms] OR Mortality [MeSH Terms] OR Frail Elderly [MeSH Terms]) AND (Humans [MeSH Terms])

#3: Systems Analysis [MeSH Terms] OR Nonlinear Dynamics [MeSH Terms] OR Fractals [MeSH Terms] OR Stochastic Processes [MeSH Terms] OR Biological Variation, Population [MeSH Terms] OR Time series analysis* OR Resilience* OR Entropy*

#4: Economic* [MeSH Terms] OR Natural Disasters [MeSH Terms] OR Geological Phenomena [MeSH Terms] OR Climatic Processes [MeSH Terms] OR Environment [MeSH Terms] OR Environmental Pollution [MeSH Terms] OR Amino Acids, Peptides, and Proteins [MeSH Terms] OR Humanities [MeSH Terms] OR Dentistry OR Dental Health Services [MeSH Terms] OR Genetic Phenomena [MeSH Terms] OR Genetic Therapy [MeSH Terms] OR Cells [MeSH Terms] OR Nurses [MeSH Terms] OR Caregivers [MeSH Terms] OR Leadership [MeSH Terms] OR Chemistry [MeSH Terms] OR Homosexuality* OR Bisexuality* OR lesbian* OR Transgender Persons* OR gay* OR Pharmacology [MeSH Terms] OR Urban Health Services [MeSH Terms] OR Socioeconomic Factors OR Sociological Factors [MeSH Terms] OR Social Behavior* OR Social Work* OR Social Problems [MeSH Terms] OR Social Environment [MeSH Terms] OR Refugees [MeSH Terms] OR Technology and Food and Beverages Category [MeSH Terms] OR Image Processing, Computer-Assisted [MeSH Terms] OR Culture [MeSH Terms] OR Radiographic Image Interpretation, Computer-Assisted [MeSH Terms] OR Image Interpretation, Computer-Assisted [MeSH Terms] OR Organization and Administration [MeSH Terms] OR Plants [MeSH Terms] OR Fungi [MeSH Terms] OR Interviews as Topic [MeSH Terms] OR Interview* OR Animal* OR Surveys Questionnaires* OR School Health Services [MeSH Terms] OR Education [MeSH Terms] OR Ergonomics [MeSH Terms] OR Osteopathic Physicians [MeSH Terms] OR Self Care[MeSH Terms] OR Stress Disorders, Traumatic [MeSH Terms] OR Population [MeSH Terms] OR Biomarkers, Tumor / blood* OR MicroRNAs / genetics* OR Child Development* OR Art Therapy [MeSH Terms] OR Nose / surgery* OR Weightlessness [MeSH Terms] OR Delphi Technique [MeSH Terms] OR Learning [MeSH Terms] OR anatomy histology* OR Veterinarians [MeSH Terms] OR Students, Health Occupations [MeSH Terms] OR Infant [MeSH Terms] OR Pandemics [MeSH Terms] OR Neoplastic Processes [MeSH Terms] OR Adolescent [MeSH Terms] OR Military Personnel [MeSH Terms] OR Bone and Bones [MeSH Terms] OR Internal-External Control [MeSH Terms] OR Rotation [MeSH Terms] OR Epidemics [MeSH Terms] OR COVID-19 [MeSH Terms] OR Cataract [MeSH Terms] OR Child [MeSH Terms]

((#1) AND (#2) AND (#3)) NOT (#4)

Filter applied:

All years

Language: English

Species: Human

Article Type included

- Case Reports
- Clinical Study
- Clinical Trial
- Comparative Study
- Controlled Clinical Trial
- Corrected and Republished Article
- Dataset
- Evaluation Study
- Guideline
- Historical Article
- Journal Article
- Meta-Analysis
- Multicenter Study
- Practice Guideline
- Pragmatic Clinical Trial
- Randomized Controlled Trial
- Review
- Systematic Review
- Twin Study
- Validation Study

Science Direct

On Science Direct, advanced searches are limited to 8 Boolean operators, so we have carried out several independent research groups (#), by putting terms to be excluded if possible.

#1: ("Adaptives capacities" OR "Adaptability" OR "Adaptation") AND ("health") AND ("complexity") AND ("fractals" OR "Nonlinear Dynamics" OR "entropy") AND ("time series")

#2: ("Adaptives capacities" OR "Adaptability" OR "Adaptation") AND ("health") AND ("Physical resilience" OR "critical slowing down") AND ("time series") NOT ("climate") NOT ("urban")

#3: ("Adaptives capacities" OR "Adaptability" OR "Adaptation") AND ("health") AND ("network physiology" OR "control theory") AND ("time series") AND ("homeostasis")

Filter applied:

All years

Article types for #1, #2 et #3 :

- Review article
- Research article
- Case reports
- Data articles
- Mini review
- Pratice guidelines
- Replication studies

Subject areas defined:

#1 :

- Medecine and Dentistry
- Engineering
- Computer science
- Neuroscience
- Psychology
- Mathematics

#2:

- Medicine and Dentistry
- Neuroscience
- Psychology
- Engineering
- Computer Science

#3 :

- Medicine and Dentistry
- Neuroscience
- Engineering
- Mathematics

Web of Science

#1: TS = (Adaptation psychological OR Adaptation physiological OR Adaptation biological OR Adaptability OR Homeostasis OR Physical resilience* OR Stability* OR Plasticity* OR Robustness* OR Biological evolution)

#2: TS = (Health OR Aging OR Elderly OR Aged OR geriatrics OR Disease OR Chronic Health Evaluation* OR Physiopathological* OR psychomotor performance OR Mortality OR Frail Elder*) AND TS =(Humans)

#3: TS = (Systems Analysis OR Nonlinear Dynamics OR Fractals OR Stochastic Processes OR Biological Variation Population OR Time series analysis* OR Resilience* OR Entropy*)

#4: TS = (Economic OR Natural Disasters OR Dentistry OR Homosexuality OR Animal OR Environmental Pollution OR Genetic OR Administration OR Child Development OR Pharmacology OR Interviews OR Plants OR Education OR Ergonomics OR Image Processing OR Socioeconomic Factors OR Social Problems OR Finance OR Chemistry OR Socioeconomic Factors OR Leadership OR Food OR Surveys Questionnaires OR protein OR cells OR climate OR CHONDROITIN SULFATE OR INHIBITOR OR molecular OR EPIDEMIC MODEL OR BIODEGRADATION OR Delay model OR nanocomposite OR BIODIVERSITY OR Air conditioning OR FISHERIES OR marine reserve OR river OR agriculture OR diet OR urban OR caries OR dentist OR newborn OR social exclusion OR Building products OR social OR DISASTER RISK REDUCTION OR human Trafficking OR Sustainable development OR grief OR HYPERINFECTIVITY OR genes OR microbiome OR fish OR migration OR neonatal OR TRANSMISSION OR MICROBIOTA OR dental OR dental implant OR supportive care OR Veterinarians OR Bones OR Adolescent OR military OR pandemics OR infant)

Filter applied:

refined by: [excluding] web of science categories: (physics mathematical or genetics heredity or chemistry analytical or electrochemistry or evolutionary biology or engineering civil or physics applied or management or nursing or ecology or polymer science or radiology nuclear medicine medical imaging or food science technology or engineering electrical electronic or chemistry applied or biotechnology applied microbiology or chemistry multidisciplinary or anthropology or environmental sciences or education educational research or materials science biomaterials or telecommunications or mechanics or physics condensed matter or computer science theory methods or reproductive biology or geosciences multidisciplinary or robotics or water resources or nanoscience nanotechnology or zoology or materials science multidisciplinary or engineering chemical or engineering mechanical or chemistry physical) and document types: (article or review) and languages: (english) and [excluding] research areas: (automation control systems or business economics or dentistry oral surgery medicine or criminology penology or crystallography or demography or agriculture or computer science or family studies or geography or information science library science or linguistics or materials science or physics or social sciences other topics or meteorology atmospheric sciences or nuclear science technology or transportation or oceanography or religion or health care sciences services or veterinary sciences or zoology)

Timespan: All years. Indexes: SCI-EXPANDED, SSCI, ESCI, IC. Timespan: all years. Indexes: sci-expanded.
